# Short-Term Association of Add-On Gosha-Jinki-Gan with Sleep and Nocturia Outcomes in Patients with Persistent Nocturia Despite Desmopressin: A Real-World Study

**DOI:** 10.3390/medicina62081466

**Published:** 2026-07-29

**Authors:** Hirofumi Kurose, Hiroshi Kishimoto, Keisuke Komiya, Kosuke Ueda, Katsuaki Chikui, Keiichiro Uemura, Kiyoaki Nishihara, Makoto Nakiri, Shigetaka Suekane, Tsukasa Igawa

**Affiliations:** 1 Department of Urology, School of Medicine, Kurume University, Kurume 830-0011, Japan; furusaka_hiro@kurume-u.ac.jp (H.K.); komiya_keisuke@kurume-u.ac.jp (K.K.); ueda_kousuke@med.kurume-u.ac.jp (K.U.); chikui_katsuaki@med.kurume-u.ac.jp (K.C.); uemura_keiichirou@kurume-u.ac.jp (K.U.); nishihara_kiyoaki@kurume-u.ac.jp (K.N.); mnakiri@med.kurume-u.ac.jp (M.N.); suekane@med.kurume-u.ac.jp (S.S.); tigawa@med.kurume-u.ac.jp (T.I.); 2 Department of Urology, Chikugo City Hospital, Chikugo 833-0041, Japan

**Keywords:** desmopressin, nocturia, Gosha-jinki-gan, sleep quality, hours of undisturbed sleep

## Abstract

*Background and Objectives:* Given the limited therapeutic options for patients with persistent nocturia despite desmopressin treatment, we aimed to evaluate the short-term effects of add-on Gosha-jinki-gan therapy on hours of undisturbed sleep (HUS) and nocturia-related parameters in a real-world clinical setting. *Materials and Methods:* This retrospective study included 40 men aged ≥60 years with nocturnal polyuria who had ≥2 nocturnal voids or HUS ≤ 3 h despite desmopressin treatment. Add-on Gosha-jinki-gan (7.5 g/day) was initiated while concomitant medications were continued. Voiding diaries and validated questionnaires were assessed before treatment and at 1 and 4 weeks. Primary outcomes were the nocturnal voiding frequency, nocturnal urine volume, nocturnal polyuria index, and HUS. Secondary outcomes included the International Prostate Symptom Score (IPSS), Overactive Bladder Symptom Score (OABSS), Athens Insomnia Scale, and Patient Global Impression of Improvement (PGI-I). *Results:* The mean voided volume before desmopressin treatment was low (109.9 mL), suggesting reduced functional bladder capacity. Add-on Gosha-jinki-gan therapy was associated with a reduction in nocturnal voiding frequency (3.38 to 2.60 episodes/night, *p* < 0.0001) and nocturnal urine volume (*p* < 0.0001). The mean HUS increased from 116 to 186 min at 4 weeks (*p* < 0.0001), representing an extension of approximately 70 min and exceeding the clinically meaningful 3 h threshold. Sleep quality, assessed by the Athens Insomnia Scale, also improved significantly. No serious adverse events were observed. *Conclusions:* Add-on Gosha-jinki-gan therapy was associated with improvements in nocturia-related parameters and prolongation of HUS in patients with persistent nocturia despite desmopressin treatment. These findings should be considered preliminary and hypothesis-generating. Prospective controlled studies are required to determine whether add-on Gosha-jinki-gan provides benefits beyond desmopressin alone.

## 1. Introduction

Nocturia, a highly prevalent lower urinary tract symptom in older adults, is associated with impaired sleep quality, an increased risk of falls, depression, frailty, and adverse health outcomes, including mortality [1]. In particular, waking to void two or more times per night significantly worsens quality of life (QOL) in older individuals [2,3]. Hours of undisturbed sleep (HUS), defined as the interval between sleep onset and the first nocturnal void, has emerged as a clinically meaningful indicator of sleep continuity and patient satisfaction [4,5]. Prolongation of HUS beyond 3 h has been shown to correlate with significant improvements in QOL among patients with nocturia [6,7].

Low-dose desmopressin effectively reduces nocturnal urine production by enhancing renal water reabsorption and has been approved for clinical use in Japan since 2019 [8,9,10]. However, a substantial proportion of patients continue to experience persistent nocturia despite adequate suppression of nocturnal urine volume [9,11]. This observation highlights the multifactorial pathophysiology of nocturia, in which bladder-related factors—such as reduced functional bladder capacity and increased afferent activity—may play an important role in addition to nocturnal polyuria.

Nocturia is increasingly recognized as a multifactorial condition involving not only nocturnal polyuria but also bladder dysfunction, sleep disturbance, and age-related physiological changes [12]. Therefore, a comprehensive therapeutic approach targeting multiple underlying mechanisms is often required in clinical practice.

In routine clinical practice, therapeutic options beyond desmopressin are limited because many patients are already receiving α1-adrenoceptor antagonists, β3-adrenoceptor agonists, or anticholinergic agents.

Gosha-jinki-gan, a traditional Japanese Kampo medicine, has long been prescribed for age-related lower urinary tract symptoms. Experimental studies suggest that it modulates the micturition reflex through inhibition of C-fiber afferent pathways and central inhibitory mechanisms without suppressing detrusor contractility [13,14,15]. These pharmacological properties indicate that Gosha-jinki-gan may target bladder-related pathophysiological components that are not directly influenced by desmopressin.

Taken together, simultaneous modulation of nocturnal urine production and bladder-related mechanisms may provide a rational therapeutic strategy for patients with persistent nocturia. Therefore, this study aimed to evaluate the short-term effects of add-on Gosha-jinki-gan therapy on HUS and nocturia-related parameters in older patients with persistent nocturia despite desmopressin treatment in a real-world clinical setting.

## 2. Materials and Methods

### 2.1. Study Design and Population

This was a single-center retrospective observational study conducted in routine clinical practice. Men aged ≥60 years who visited our institution with nocturia as the chief complaint were screened for eligibility. Patients were included if their 3-day bladder diary demonstrated a mean nocturnal voiding frequency of ≥2 episodes per night and a mean nocturnal polyuria index (NPi; nocturnal urine volume/24 h urine volume × 100%) of ≥33%, and desmopressin (Minirin Melt^®^ OD Tablet; Ferring Pharmaceuticals Co., Ltd., Tokyo, Japan) therapy had been initiated for nocturnal polyuria.

Among these patients, those who continued to experience persistent nocturia despite desmopressin treatment—defined as ≥2 nocturnal voids per night or ≤3 h of undisturbed sleep (HUS) on follow-up bladder diary assessment—were eligible for add-on Gosha-jinki-gan therapy. A total of 40 consecutive patients who met these criteria were included in the analysis.

Patients were retrospectively identified from routine clinical practice based on the predefined eligibility criteria. Because the study was not originally designed as a prospective research protocol, the total number of patients screened during the study period could not be reliably determined.

Patients were excluded if desmopressin was contraindicated according to the package insert. Contraindications included hyponatremia, habitual or psychogenic polydipsia, heart failure, syndrome of inappropriate antidiuretic hormone secretion, current treatment with diuretics or systemic corticosteroids, moderate-to-severe renal impairment, or known hypersensitivity to desmopressin or Gosha-jinki-gan.

### 2.2. Treatment Protocol

In accordance with Japanese clinical guidelines for nocturia management, behavioral interventions, optimization of pharmacological treatment for bladder storage dysfunction, and management of sleep disorders were implemented prior to desmopressin initiation. Desmopressin was initiated at a starting dose of 50 μg in patients with nocturnal polyuria-associated nocturia.

In patients who continued to experience ≥2 nocturnal voids per night or <3 h of HUS despite desmopressin treatment, add-on therapy with Gosha-jinki-gan (Tsumura & Co., Tokyo, Japan) was initiated at a dose of 7.5 g/day, administered in three divided doses.

Concomitant medications for lower urinary tract symptoms—including α1-adrenoceptor antagonists, phosphodiesterase type 5 inhibitors, 5α-reductase inhibitors, anticholinergic agents, and β3-adrenoceptor agonists—were continued without modification during the add-on treatment period. The majority of patients were receiving concomitant pharmacological therapy, reflecting real-world clinical practice.

### 2.3. Outcome Measures

Efficacy outcomes were evaluated at three time points: before desmopressin treatment (baseline), immediately before initiation of Gosha-jinki-gan therapy, and 4 weeks after the start of add-on therapy. Data were obtained from standardized 3-day bladder diaries routinely used in clinical practice.

The primary endpoints were nocturnal voiding frequency, nocturnal urine volume, nocturnal polyuria index, and HUS. Additional bladder diary parameters included first nocturnal voided volume and total daily voiding frequency.

Patient-reported outcomes were assessed using the International Prostate Symptom Score (IPSS), Overactive Bladder Symptom Score (OABSS), and Athens Insomnia Scale. Global subjective improvement at 4 weeks was evaluated using the Patient Global Impression of Improvement (PGI-I).

All bladder diaries were reviewed and confirmed by attending urologists to ensure accuracy. Patients were instructed on how to complete the diaries prior to assessment. Adherence to Gosha-jinki-gan treatment was assessed based on patient self-report at follow-up visits.

### 2.4. Statistical Analysis

Statistical analyses were performed using JMP version 17.0 (SAS Institute Inc., Cary, NC, USA). Within-group comparisons were conducted using the Wilcoxon signed-rank test. Between-group comparisons were performed using the Mann–Whitney U test where appropriate. Correlations were assessed using Spearman’s rank correlation coefficient. A two-sided *p*-value < 0.05 was considered statistically significant.

### 2.5. Ethical Approval

This study was approved by the Ethics Committee of Chikugo City Hospital (approval number: 2025-04). The requirement for written informed consent was waived due to the retrospective study design, and an opt-out approach was adopted.

## 3. Results

### 3.1. Patient Characteristics

A total of 40 consecutive patients were included in the present analysis. Baseline characteristics are summarized in Table 1. The mean age was 78.9 years, indicating an elderly study population, and the mean body mass index was 23.9 kg/m^2^. Prior to desmopressin treatment, 85% of patients were already receiving pharmacological therapy for lower urinary tract symptoms other than desmopressin. The mean duration of desmopressin treatment before initiation of add-on Gosha-jinki-gan therapy was 473 days, indicating persistent symptoms despite long-term treatment.

### 3.2. Efficacy Outcomes

#### Bladder Diary Parameters

**Figure 1:**
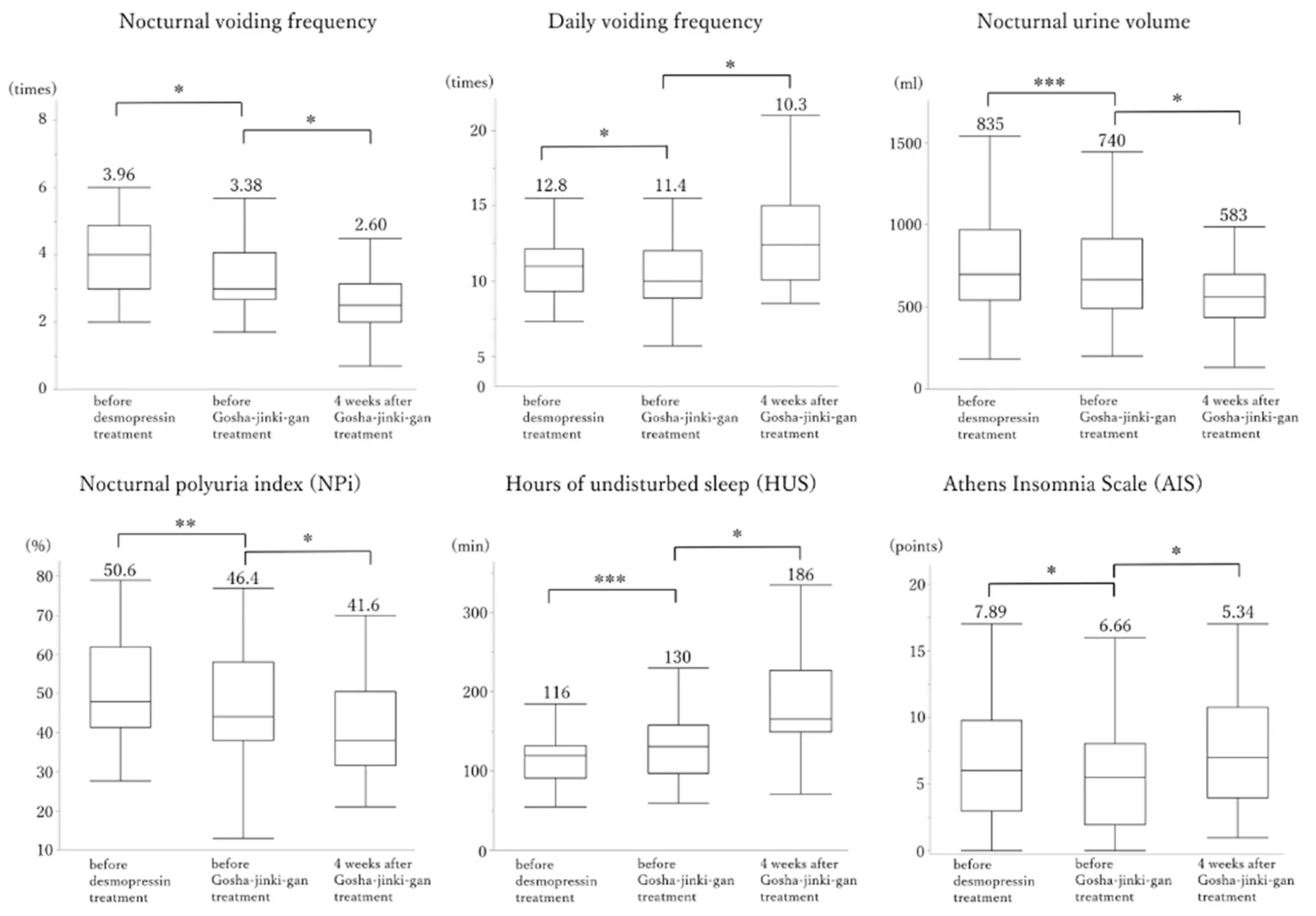
Changes in bladder diary parameters at three time points: before desmopressin treatment (baseline), immediately before initiation of add-on Gosha-jinki-gan therapy (pre–GJG), and 4 weeks after initiation of add-on therapy (4 weeks). Data are presented as mean ± standard deviation (SD). Comparisons were performed using the Wilcoxon signed-rank test. * *p* < 0.05; ** *p* < 0.01; *** n.s., not significant.

Changes in bladder diary parameters are presented in Figure 1.

Nocturnal voiding frequency significantly decreased after desmopressin treatment (from 3.96 to 3.38 episodes per night, *p* = 0.004). Following the initiation of add-on Gosha-jinki-gan therapy, nocturnal voiding frequency further decreased significantly to 2.60 episodes per night at 4 weeks (*p* < 0.0001).

Total daily voiding frequency also significantly decreased during the study period, with reductions observed between baseline and immediately before add-on therapy (*p* = 0.0008), and between pre–Gosha-jinki-gan and 4 weeks after initiation (*p* = 0.0003).

Nocturnal urine volume showed a decreasing trend after desmopressin treatment; however, this change did not reach statistical significance (*p* = 0.129). In contrast, add-on Gosha-jinki-gan therapy resulted in a significant reduction in nocturnal urine volume (*p* < 0.0001). Similarly, the nocturnal polyuria index (NPi) significantly decreased both between baseline and pre-Gosha-jinki-gan (*p* = 0.045) and between pre-treatment and 4 weeks (*p* = 0.0008).

The duration of undisturbed sleep (HUS) significantly increased during the study period. Mean HUS increased from 116 min at baseline to 186 min at 4 weeks after initiation of add-on therapy (*p* < 0.0001), representing an extension of approximately 70 min and surpassing the clinically meaningful 3 h threshold in a substantial proportion of patients. In parallel with the prolongation of HUS, Athens Insomnia Scale scores significantly improved both after desmopressin treatment (*p* = 0.009) and following add-on therapy (*p* = 0.0003).

The mean voided volume before desmopressin treatment was 109.9 mL, suggesting reduced functional bladder capacity at baseline.

### 3.3. Questionnaire-Based Outcomes

**Figure 2:**
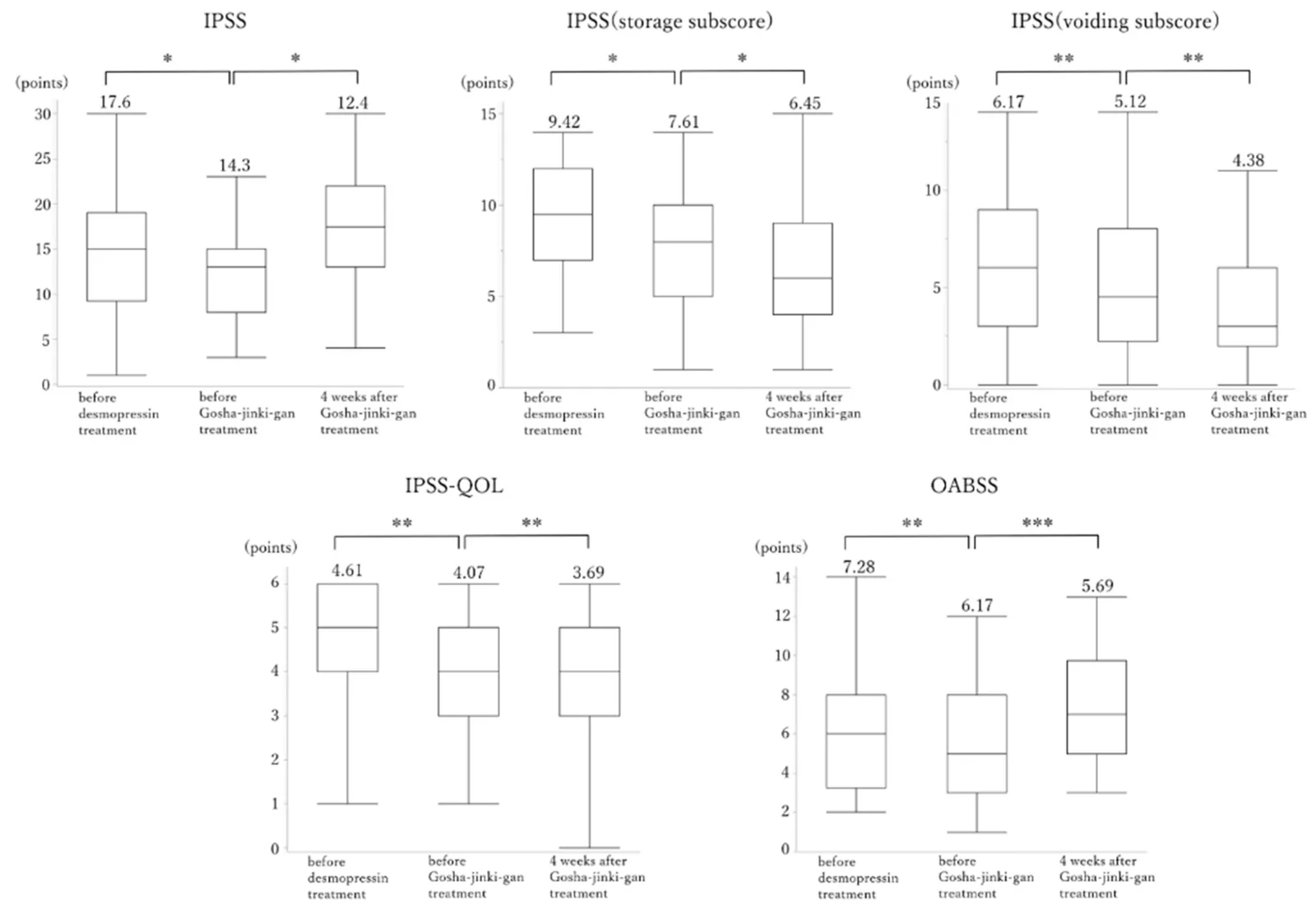
Changes in questionnaire-based scores before add-on Gosha-jinki-gan therapy and 4 weeks after treatment. Data are presented as boxplots showing the median, interquartile range, and range. Numerical values indicate mean scores. IPSS, International Prostate Symptom Score; IPSS-QoL, International Prostate Symptom Score Quality of Life Index; OABSS, Overactive Bladder Symptom Score. (* *p* < 0.01, ** *p* < 0.05, *** n.s., not significant).

Changes in patient-reported outcome measures are shown in Figure 2.

The International Prostate Symptom Score (IPSS) significantly improved 4 weeks after initiation of add-on Gosha-jinki-gan therapy compared with pre-treatment values (*p* = 0.003). The IPSS QOL score also significantly improved (*p* = 0.034). Both the storage symptom subscore (*p* = 0.002) and voiding symptom subscore (*p* = 0.016) showed significant reductions at 4 weeks.

The Overactive Bladder Symptom Score (OABSS) showed a trend toward improvement, although this did not reach statistical significance (*p* = 0.079).

Global subjective assessment using the Patient Global Impression of Improvement (PGI-I) at 4 weeks revealed that 18.4% of patients reported “very much improved,” 39.4% “much improved,” and 23.7% “minimally improved,” whereas 18.4% reported “no change.” No patient reported worsening of symptoms (score ≥ 5) (Table 2).

### 3.4. Safety Outcomes

Regarding safety, two patients discontinued Gosha-jinki-gan therapy because of grade 1 diarrhea, and one patient discontinued treatment because of difficulty with medication intake. The treatment continuation rate at 4 weeks was 92.5%. No serious adverse events were observed during the study period.

## 4. Discussion

This study suggests that add-on Gosha-jinki-gan therapy was associated with improvements in nocturia-related parameters and prolongation of hours of undisturbed sleep (HUS) in patients with persistent nocturia despite desmopressin treatment. Undisturbed sleep was prolonged by approximately 60 min, with a mean HUS exceeding 180 min at 4 weeks. Achievement of undisturbed sleep beyond 3 h may represent not merely an increase in total sleep duration but also an improvement in sleep continuity, particularly during the early nocturnal period when slow-wave sleep predominates [16]. In our previous studies, improvements in QOL and sleep satisfaction were more strongly related to prolongation of HUS than to reduction in nocturnal voiding frequency [6]. The present findings suggest that add-on therapy may contribute to achieving the clinically meaningful “3 h HUS threshold” in patients who remain symptomatic despite desmopressin. This concept is consistent with the International Continence Society consensus, which recognizes the close relationship between nocturia, sleep disturbance, and health-related quality of life and emphasizes the importance of clinically meaningful outcomes beyond nocturnal voiding frequency alone [12]. Although the magnitude of improvement was modest, the study population consisted of patients with persistent symptoms despite long-term desmopressin treatment, a clinical setting in which even incremental improvements may be meaningful.

Desmopressin reduces nocturnal urine production through enhanced renal water reabsorption but does not directly modulate bladder function [8,9]. Persistent nocturia despite adequate suppression of urine volume suggests that bladder-related mechanisms—such as reduced functional bladder capacity and increased afferent signaling—play a critical role in a subset of patients. In the present cohort, prolonged desmopressin treatment failed to provide sufficient symptom control, supporting the concept of multifactorial pathophysiology in nocturia.

Experimental studies have shown that Gosha-jinki-gan modulates the micturition reflex without suppressing detrusor contractility, likely through inhibition of C-fiber afferent pathways and modulation of central inhibitory mechanisms [14,15]. Although Gosha-jinki-gan has primarily been reported to modulate bladder afferent activity, the present study also demonstrated significant reductions in nocturnal urine volume and the nocturnal polyuria index. The mechanism underlying these findings remains unclear. However, previous studies have suggested that Gosha-jinki-gan may exert systemic effects beyond the lower urinary tract. Therefore, both bladder-related and urine production-related mechanisms may have contributed to the observed prolongation of HUS. Further mechanistic studies are warranted to clarify these effects.

Thus, combination therapy with desmopressin and Gosha-jinki-gan may target complementary pathophysiological domains related to nocturia, providing a plausible explanation for the observed prolongation of HUS.

Patients with small voided volumes are particularly susceptible to early awakening, even when nocturnal urine production is partially controlled. The low baseline mean voided volume (109.9 mL) in this cohort suggests a reduced-capacity bladder phenotype. In such patients, bladder-related mechanisms may predominate over urine volume as drivers of nocturia. Add-on therapy may therefore be particularly relevant in this phenotype, although causal relationships cannot be established in this retrospective study.

From a clinical perspective, the present findings may support a stepwise treatment strategy in patients with persistent nocturia despite desmopressin, particularly in those with suspected reduced functional bladder capacity. Incorporating therapies that target bladder-related mechanisms may help optimize treatment outcomes in real-world practice. Furthermore, this approach may be particularly applicable in elderly patients receiving multiple pharmacological treatments, in whom therapeutic options are often limited.

Safety is an important consideration in elderly patients with nocturia, who frequently receive multiple medications. In this study, add-on Gosha-jinki-gan therapy was well tolerated, with a high continuation rate (92.5%) and no serious adverse events. Mild diarrhea and difficulty with medication intake led to treatment discontinuation in a small number of patients. Because Gosha-jinki-gan does not impair detrusor contractility, the risk of urinary retention appears low; however, further studies are needed to confirm long-term safety. In addition, Gosha-jinki-gan is widely available and covered by the Japanese national health insurance system, resulting in relatively low patient costs and potentially enhancing its practicality in routine clinical practice.

This study has several limitations. First, it was a single-center retrospective analysis with a relatively small sample size, and the observation period was limited to 4 weeks. Second, the absence of a control group precludes definitive conclusions regarding causality, and the findings should therefore be considered hypothesis-generating. Third, information regarding underlying pathophysiological conditions contributing to nocturia, such as neuropathic disorders, neurogenic bladder, and bladder outlet obstruction, was not systematically collected. Therefore, treatment responses according to specific etiologies could not be evaluated. In addition, only male patients were included in this study; therefore, the generalizability of the findings to female patients remains uncertain. Furthermore, laboratory parameters including renal function, serum electrolytes, glucose levels, and urinalysis findings were not collected in a standardized manner and were therefore unavailable for retrospective analysis. In addition, sleep outcomes were assessed using questionnaires and bladder diaries rather than objective measurements such as polysomnography. Given these limitations, the findings should be interpreted with caution. Prospective randomized controlled studies comparing add-on Gosha-jinki-gan therapy, Gosha-jinki-gan monotherapy, and placebo are required to confirm these observations and clarify the independent contribution of Gosha-jinki-gan to treatment outcomes.

## 5. Conclusions

In conclusion, in this real-world retrospective study, add-on Gosha-jinki-gan therapy was associated with improvements in nocturia-related parameters and prolongation of HUS in patients with persistent nocturia despite desmopressin treatment. Given the absence of a control group, these findings should be considered preliminary and hypothesis-generating. Add-on Gosha-jinki-gan therapy may warrant further evaluation in prospective controlled studies, particularly in patients with reduced functional bladder capacity. Future studies should explore patient phenotypes that may benefit most from such combination therapy.

## Figures and Tables

**Table 1:** Patient Characteristics.

Patient Characteristics	
Patients, no	40
Age, years	78.9 ± 6.84
Weight, kg	63.9 ± 10.4
Body Mass Index, kg/m^2^	23.9 ± 3.44
Hypertension, *n* (%)	34 (85.0%)
History of diabetes mellitus, *n* (%)	13 (32.5%)
History of dyslipidemia, *n* (%)	17 (42.5%)
Prostate volume, mL	37.3 ± 17.1
Postvoid residual volume, mL	39.2 ± 33.4
History of LUTS medication use, *n* (%)	34 (85.0%)
α1-blockers, *n* (%)	22 (55.0%)
β3-agonists, *n* (%)	31 (77.5%)
Duration of desmopressin treatment, days	473 ± 450
Voided volume before desmopressin treatment, mL	109.9 ± 51.2

**Table 2:** Patient Global Impression of Improvement (PGI-I) after 4 Weeks of Gosha-jinki-gan Treatment.

Score		
1	Very much better	7 (18.4%)
2	Much better	15 (39.4%)
3	A little better	9 (23.7%)
4	No change	7 (18.4%)
5	A little worse	0 (0.0%)
6	Much worse	0 (0.0%)
7	Very much worse	0 (0.0%)

## Data Availability

The datasets generated and/or analyzed during the current study are available from the corresponding author on reasonable request.
